# Ensemble yield simulations: Using heat-tolerant and later-maturing varieties to adapt to climate warming

**DOI:** 10.1371/journal.pone.0176766

**Published:** 2017-05-01

**Authors:** Yi Zhang, Yanxia Zhao

**Affiliations:** 1 State Key Laboratory of Severe Weather, Chinese Academy of Meteorological Sciences, Beijing, China; 2 Shanghai Institute of Meteorological Sciences, Shanghai, China; Agroecological Institute, CHINA

## Abstract

The use of modern crop varieties is a dominant method of obtaining high yields in crop production. Efforts to identify suitable varieties, with characteristics that would increase crop yield under future climate conditions, remain essential to developing sustainable agriculture and food security. This work aims to evaluate potential genotypic adaptations (i.e., using varieties with increased ability to produce desirable grain numbers under high temperatures and with enhanced thermal time requirements during the grain-filling period) to cope with the negative impacts of climate change on maize yield. The contributions of different options were investigated at six sites in the North China Plain using the APSIM model and the outputs of 8 GCMs under RCP4.5 scenarios. It was found that without considering adaptation options, mean maize yield would decrease by 7~18% during 2010–2039 relative to 1976–2005. A large decrease in grain number relative to stabilized grain weight decreased maize yield under future climate scenarios. Using heat-tolerant varieties, maize yield could increase on average by 6% to 10%. Using later maturing varieties, e.g., enhanced thermal time requirements during the grain-filling period, maize yield could increase by 7% to 10%. The optimal adaptation options were site specific.

## Introduction

The negative impact of climate warming on food crops has been reported frequently and could pose a significant challenge to food security in China [1–3]. According to the “national food security and long-term planning framework,” the nation’s food quantity should increase 5×10^10^ kg until 2020. Improving understanding about effective climate change adaptation options to increase crop yields is essential to develop sustainable agriculture and food security. Recent practices show that modern varieties have dramatically increased crop yield [4–6], which have proven to also be suitable to conditions of rising temperatures in different regions of the world. Efforts to identify suitable varieties with the characteristics of increasing crop yield would be important to the development of sustainable agriculture for a future climate. Furthermore, projections of future climate change are inherently uncertain, given the different characteristics of GCMs, and this must also be considered when evaluating climate change impacts [7,8]. In our study, the ensemble method was used to address this uncertainty [9,10].

**Fig 1:**
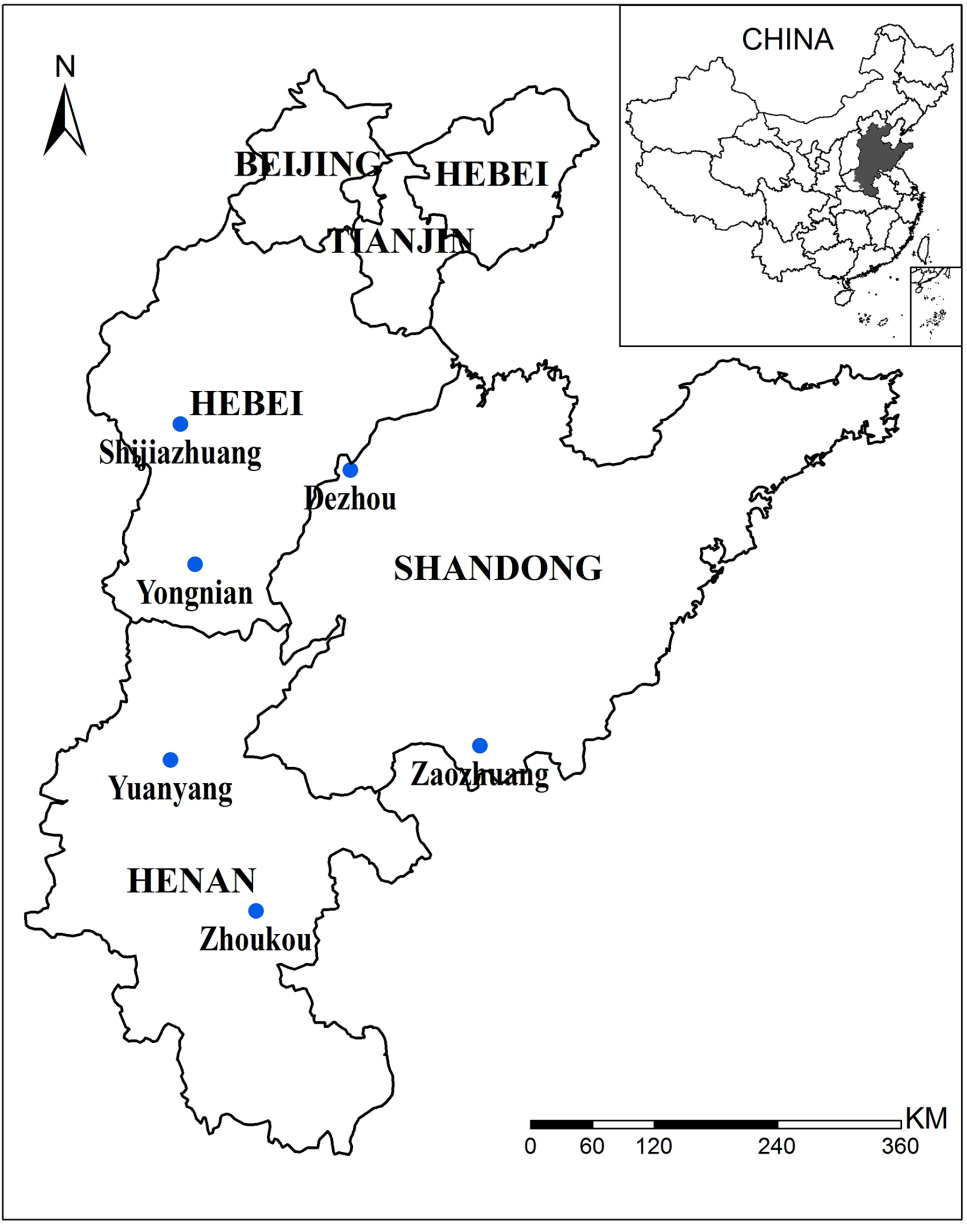
Locations of study sites in the North China Plain.

The North China Plain (NCP) is the largest summer maize production area in China (Fig 1). Maize experiences a hot summer growing season and heat stress, particularly during reproduction, which could dramatically decrease yields in the NCP [11,12]. With global warming, extremely high temperatures and heat stress are likely to become more frequent, constituting an important threat to global food supply [13,14]. Using varieties with improved heat tolerance and cardinal temperatures is a successful strategy to cope with heat stress [11,15]. Much experimental evidence has demonstrated the relationship between high temperatures in the period centered on flowering and reduced grain number, with significant negative impacts on grain yield [16,17]. However, the adoption of varieties with improved high-temperature tolerance of pollen viability, e.g., formation of grain number, which is the determining factor in maize yield [18], has occurred much more slowly. Furthermore, “Double-Delay” technology on wheat-maize cropping systems in the NCP has been proven to be a valid planned adaptation strategy to climate warming in recent years [19,20]. The delayed sowing of wheat allows a longer duration of maize growth (longer grain filling period) or accommodation of later-maturing maize varieties. Improved understand of whether using a later-maturing maize variety could contribute to an increase in crop yield is essential to the development of an adaptation strategy against future climate warming.

In this context, we use synthetic maize varieties characterized by improved high-temperature tolerance of grain number formation and enhanced thermal time requirements during the grain-filling period in the APSIM model to investigate the potential impact of genotypic adaptation to future climate scenarios. To account for the uncertainty of climate projections, the APSIM model was linked with the outputs of 8 GCMs under the RCP4.5 scenarios.

## Materials and methods

### Study sites

Six study sites, including Yuanyang (YY) and Zhoukou (ZK) in Henan Province, Yongnian (YN) and Shijiazhuang (SJZ) in Hebei Province, and Zaozhuang (ZZ) and Dezhou (DZ) in Shandong Province were selected (Fig 1) because they are located in the major maize production area in the NCP, and high-quality data are available for the maize variety *Xundan29*, including the phenological stages of flowering and maturity, grain yield and management practices. The data were collected from the regional test of maize varieties in the Huanghuaihai area during 2008–2009 (S1 Table).

### Data of GCM simulations

Outputs from 8 GCMs under the RCP4.5 scenario archived by CMIP5 were used in the present study: BCC-CSM1-1 (China), CCSM4 (USA), CSIRO-MK3-6-0 (Australia), EC-EARTH (Europe), GFDL-ESM2G (USA), IPSL-CM5A-MR (France), MRI-CGCM3 (Japan), and NorESM1-M (Norway). Detailed information on forcing data can be found on the CMIP5 website (http://cmip-pcmdi.llnl.gov/cmip5/availability.html). Many previous studies have demonstrated that the uncertainty from the CO_2_ effect is smaller than that from climate in the yield prediction, especially for C4 crop [21,22]. As a consequence, only RCP4.5, which refers to a radiative forcing stabilized at 4.5 W m^-2^ by the year 2100, was selected in the study. All GCM outputs were interpolated bilinearly to a common resolution of 1.0°× 1.0° grids. The time slices of 1976–2005 and 2010–2039 were selected to represent the baseline and future climate, respectively.

Table 1 summarizes the increases of maximum and minimum temperature during 2010–2039, with reference to the maize-growing season (June-September) during 1976–2005. The mean maximum temperature generally increased from 0.4°C to 1.2°C under RCP4.5 during the period 2010–2039 at all sites relative to 1976–2005. The increases in mean minimum temperature were greater under the future scenarios than those of the mean maximum temperature at all sites, which were 0.7–1.4°C under RCP4.5.

**Table 1:** Average difference between baseline (1976–2005) and future period (2010–2039) in RCP4.5 for maximum (T_max_) and minimum (T_min_) temperature under 8 GCMs during maize growth.

Site	ΔT_min_ (°C)/Month				ΔT_max_ (°C)/Month			
	Jun	Jul	Aug	Sep	Jun	Jul	Aug	Sep
Yuanyang	1.1±0.2	0.9±0.3	1.0±0.2	1.3±0.3	0.6±0.2	0.5±0.3	0.6±0.1	0.8±0.2
Zhoukou	1.0±0.3	0.7±0.1	0.6±0.3	1.1±0.1	0.5±0.2	0.7±0.2	0.9±0.3	1.1±0.1
Zaozhuang	0.9±0.2	0.7±0.3	0.7±0.2	1.0±0.3	0.6±0.3	0.5±0.3	0.8±0.3	0.9±0.1
Dezhou	1.0±0.3	1.1±0.2	0.9±0.2	1.3±0.1	0.7±0.1	0.7±0.2	0.6±0.1	1.2±0.2
Yongnian	0.7±0.3	1.0±0.4	1.0±0.3	1.0±0.3	0.4±0.1	0.5±0.2	0.5±0.3	0.7±0.3
Shijiazhuang	0.9±0.2	1.0±0.1	0.5±0.2	1.4±0.2	0.5±0.2	0.6±0.1	0.5±0.3	1.1±0.2

The standard deviation of the mean is shown.

### The APSIM model and its evaluation

APSIM [23,24] was used to simulate crop yield as a function of current and future climate conditions. This model has proven to be an effective tool for reproducing observed crop growth and yield and for investigating the impacts of climate variability on crop productivity in the NCP [20,25,26].

Prior to its use, using a trial-and-error method, APSIM-Maize was calibrated based on measured flowering, maturity and maize yield at all sites in 2008 to determine the parameters for the variety *Xundan29*. The parameters included thermal time required from emergence to end of the juvenile stage (143°Cd), thermal time required from flowering to maturity (850°Cd), photoperiod slope (18°Ch^-1^), potential grain numbers per head (600), grain-filling rate (11 mg of grain per day), and maximum temperature above which grain number is reduced (36°C). The calibrated model was then evaluated using experimental data from 2009. The coefficient of determination (*R*^*2*^) of the original regression lines and the root mean square errors (*RMSE*) between the simulated and measured values were calculated to evaluate model performance.

### Simulations and analysis

All the simulations were run by the APSIM model and the outputs of 8 GCMs for climate change period 2010–2039 and compared with the corresponding simulation under baseline climate conditions (1976–2005). To provide an ensemble effect of climate change on maize yield changes under 8 GCMs, probability density functions (PDFs) and cumulative distribution functions (CDFs) were used to describe their probability distributions.

One synthetic maize variety with improved high-temperature tolerance to grain formation was expressed in the APSIM model. The high-temperature stress function introduced in the model to describe the response of grain-number to temperature is [27]:

$$\delta =1-0.019(T_{\text{max}}-T_{\text{lim}})$$

Grain-number fertility percentage (*δ*) reduced when the daily maximum temperature (*T*_*max*_, °C) exceeded a threshold temperature (*T*_*lim*_, °C) around anthesis. For the variety *Xundan29*, *T*_*lim*_ had a value of 36°C. To simulate the possible effect of an increase in tolerance of grain number to high temperatures, we assumed that this response would be shifted, increasing the value of *T*_*lim*_ to 37°C.

The other synthetic maize variety, with an enhanced grain-filling period, was represented by enhancing the coefficient of thermal time required from flowering to maturity used by the model. For the variety *Xundan29*, the coefficient of thermal time required from flowering to maturity is 850°Cd. To simulate the possible effect of the required thermal time, we assumed that this value would be increased to 950°Cd.

## Results

### APSIM-Maize performance

**Fig 2:**
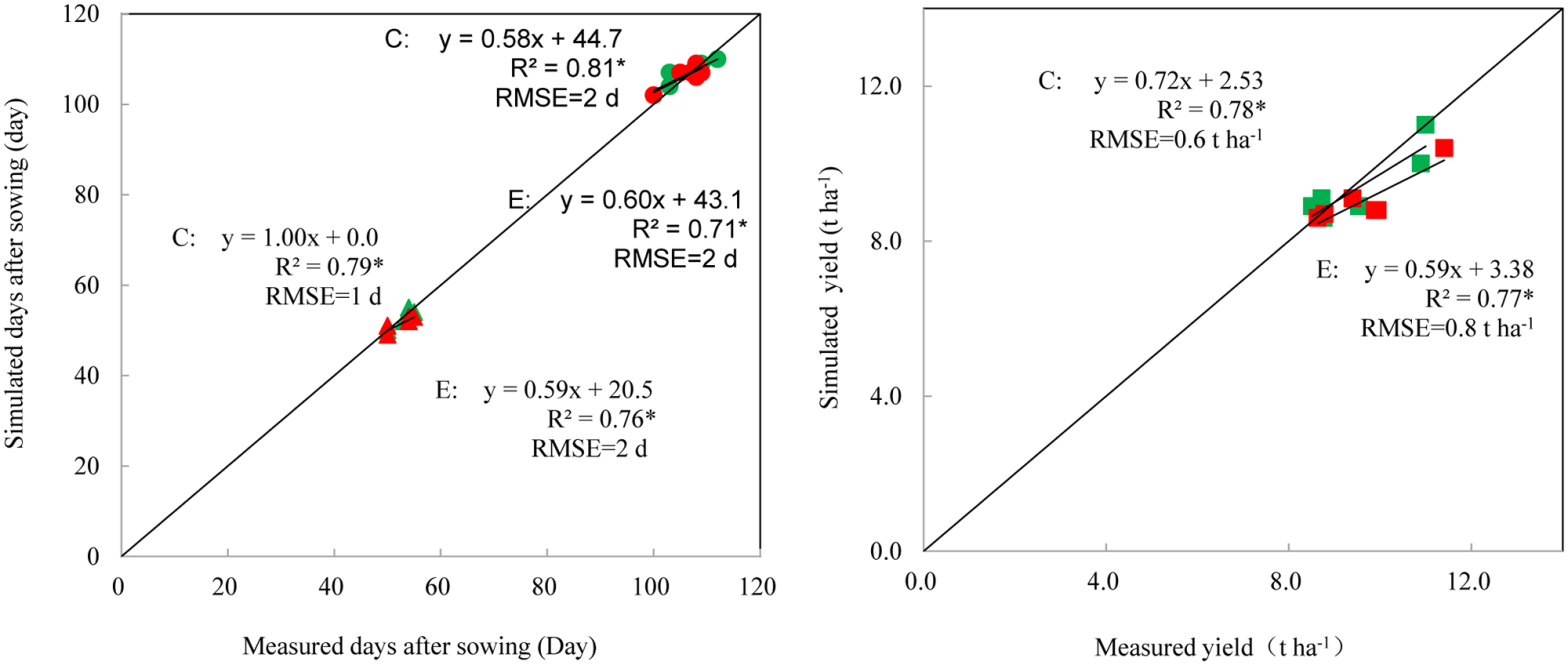
The calibration and evaluation results between observed and simulated flowering (Δ), maturity (○) and yield (□) of maize. C represents the calibration results of green symbols. E represents the evaluation results of red symbols. RMSE represents the absolute root mean square errors. Significant at *P<0.05.

Fig 2 compares the simulated and measured maize phenology and yield values for the variety *Xundan29* based on the different study sites. The simulated dates of flowering and maturity were close to the observed values. Grain yield was reasonably well predicted. Overall, more than 70% of the variation in the measured phenology and grain yield was explained by the model. The RMSE of simulated flowering and maturity dates was 1 and 2 days, respectively, with average error of 3% for the flowering date and 2% for the maturity date. The RMSE of simulated maize yields was 0.6–0.8 t ha^-1^, with errors generally less than 10%.

### Climate change impacts

**Fig 3:**
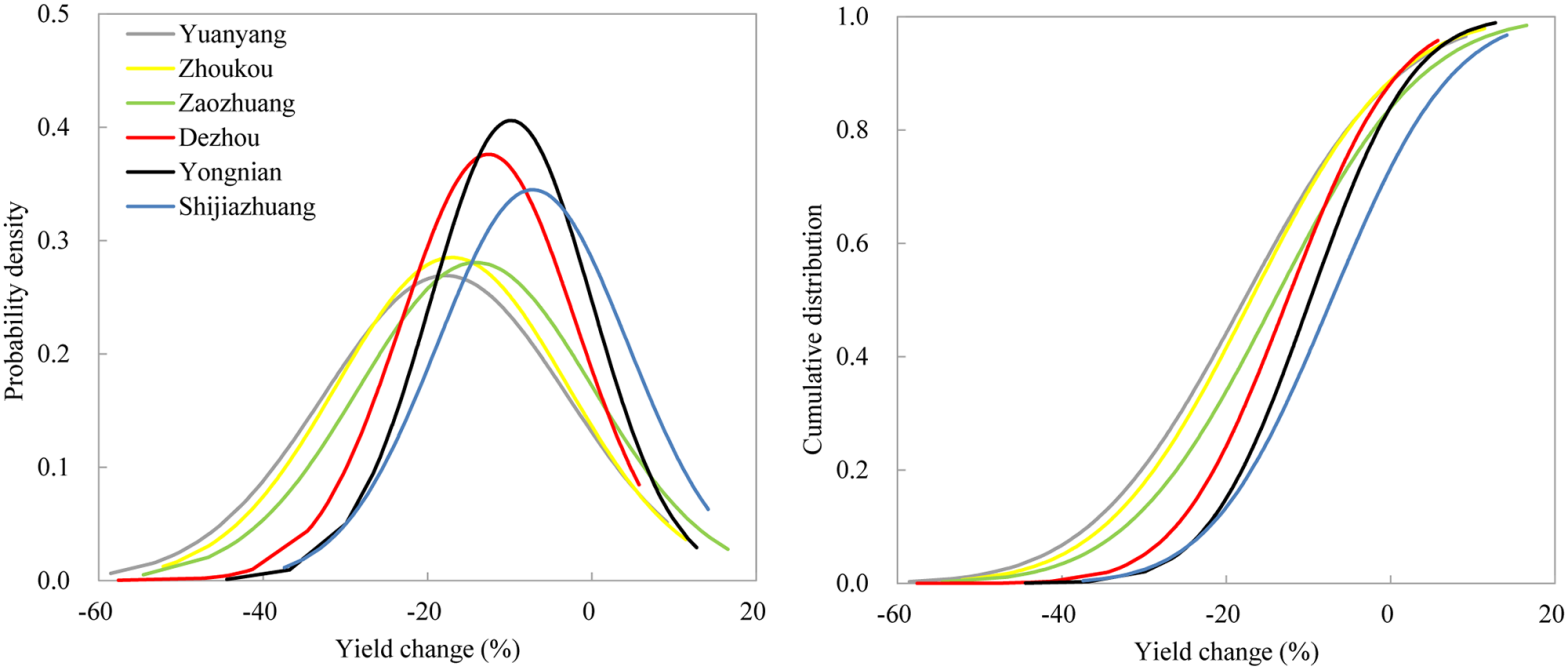
PDFs and CDFs of yield changes for variety *Xundan29*.

The PDFs and CDFs of the simulated yield changes are presented in Fig 3. All PDFs of yield changes shift more negatively, and the negative tail widens from SJZ to YY. In general, the mean projected decreases in maize yield ranged from 7% at SJZ to 18% at YY. The highest probability at all sites is a yield change in the –20% to 0% interval. However, the probabilities of yield change in the –40% to –20% and –20% to 0% intervals are approximately the same at YY and ZK. All CDFs showed that the probability of maize yield decrease was more than 70%.

**Fig 4:**
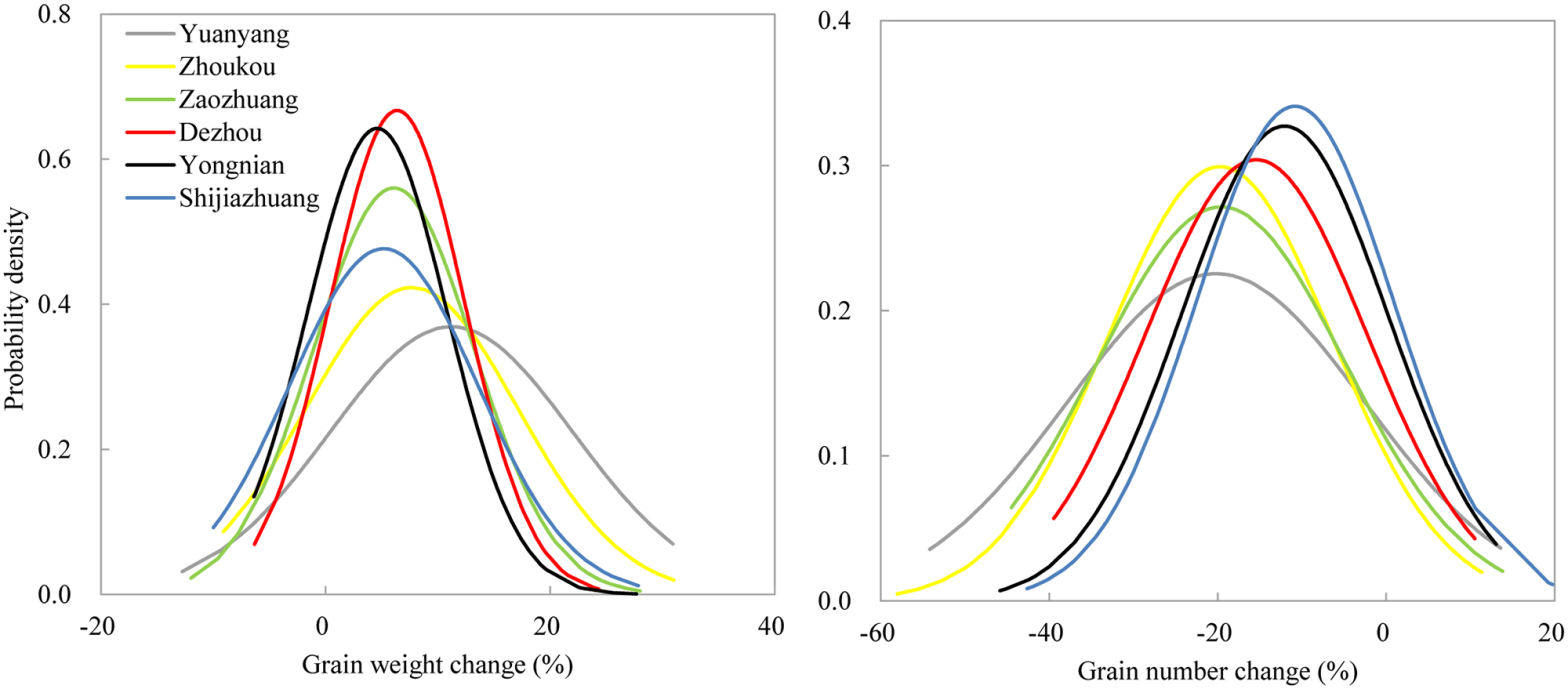
PDFs of grain weight and number changes for variety *Xundan29*.

The changes in yield components are shown in Fig 4. Interestingly, the results suggest increased grain weight but a significant decrease in grain number during grain development. The projection for grain weight increased by 5% (SJZ) ~ 11% (YY), with the probability of 73% (SJZ) ~ 86% (DZ) in the period 2010–2039. The reduction of grain number was large, with the percentages relative to the historical value of 11% (SJZ) ~ 20% (YY). The CDFs point to a probable decrease in maize grain number, ranging from 83% at SJZ to 94% at ZK in the period 2010–2039. Therefore, the large decreases in maize yield were related to the decreases in grain number caused by supra-optimal temperatures around flowering.

### Adaptation options

#### A heat-tolerant variety

**Fig 5:**
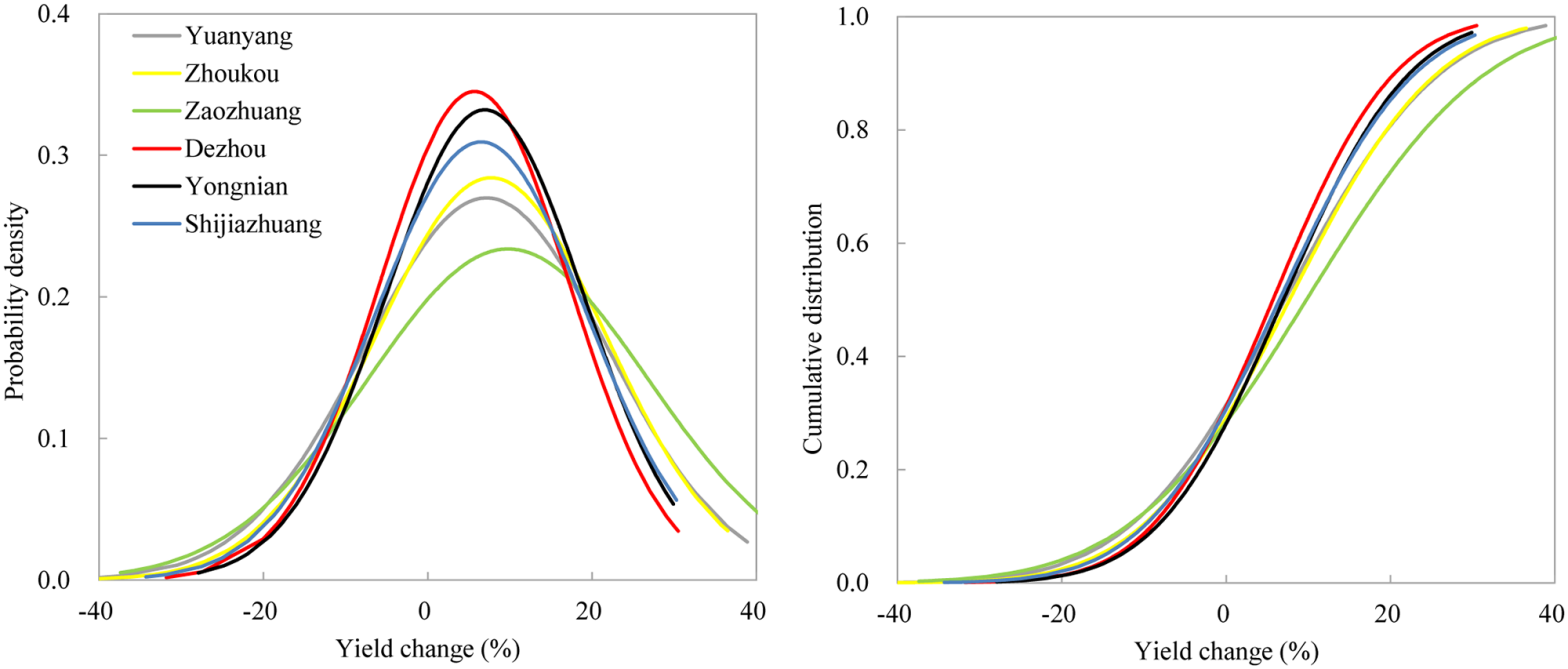
PDFs and CDFs of yield changes for variety *Xundan29* with improved temperature tolerance.

The results of using a synthetic variety with improved tolerance of grain number formation to high temperature are given in Fig 5. The mean of the PDFs shift more to the positive side, indicating the relatively large possibility of yield increases. Comparing the PDFs, we can see that the dispersions of yield changes were relatively stable. The maize yield would be increased by 6% (DZ) ~ 10% (ZZ), with an S.D. range of 12% (DZ) ~ 17% (ZZ). The yield change in the 0% to 20% interval has the maximum probability at all stations. The CDFs showed that the maize yield would increase with a probability of 65% (YY) ~ 73% (DZ).

#### A longer grain-filling variety

**Fig 6:**
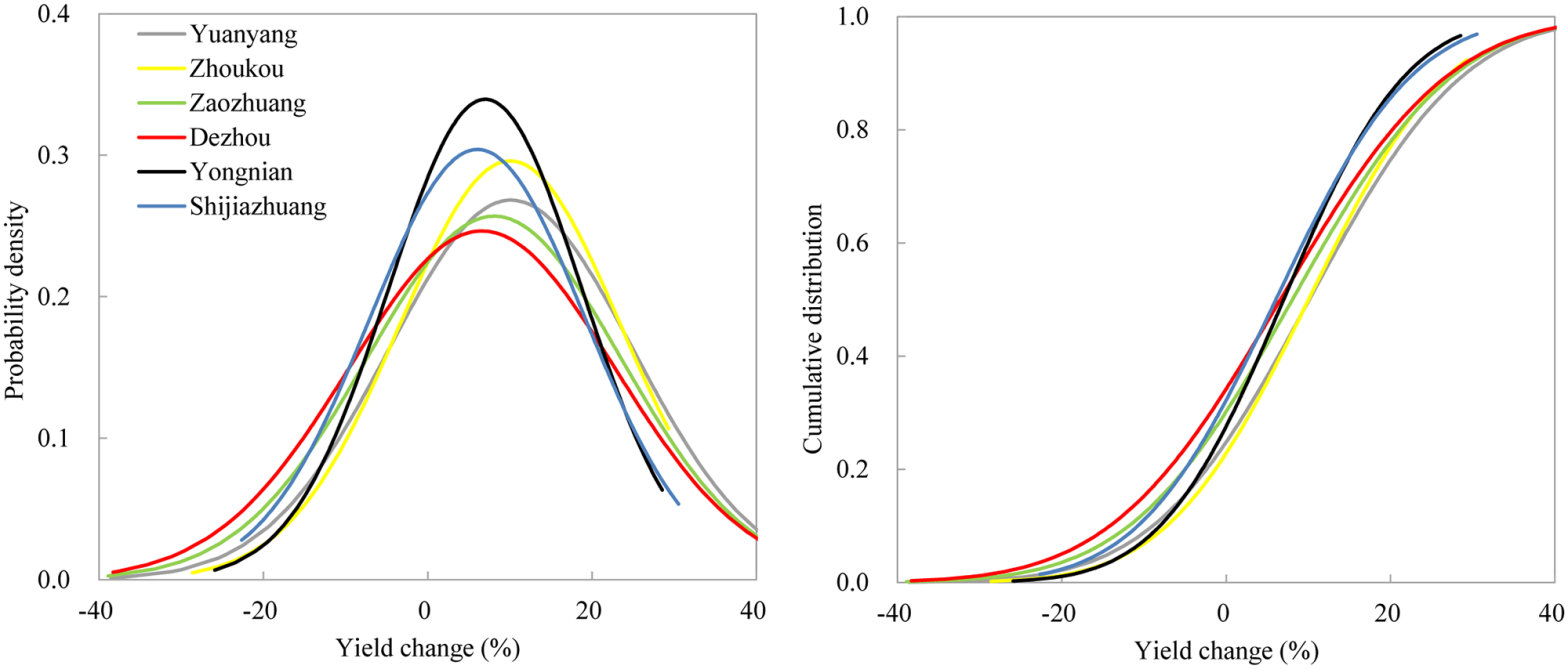
PDFs and CDFs of yield changes for variety *Xundan29* with enhanced grain-filling period.

The results of using a synthetic variety with an enhanced grain-filling period are presented in Fig 6. The peak of the PDFs indicated that the variety had beneficial impacts on yield increases, which ranged from 7% at SJZ to 10% at YY. Compared with Fig 3, yield change with intervals of –60% ~ –40% were absent. The CDFs suggested that maize yield would increase, with a probability of 66% (DZ) ~ 78% (ZK).

## Discussion

In the present study, we assessed the impacts of climate change on maize yield and yield components in NCP while accounting for climate uncertainty. Like many previous studies [28,29], a decline in maize yield was found in our study. Furthermore, a large decrease in grain number relative to the stabilized grain weight decreased maize yield under the future climate scenarios in our study. Maize grain number is very sensitive to environmental stresses, such as high temperature around flowering, and strongly associated with final yield [30–32]. This mechanism is observed in both controlled environments and field studies [33–35].

One simple genotypic adaptation to the future climate explored in the study can be obtained through enhanced thermal time requirements during the grain-filling period, mimicking later maturing varieties. It should be noted that in this study, the benefit to maize of using later-maturing varieties did not consider wheat production in the wheat-maize cropping system in the NCP. Actually, “Double-Delay” technology considers the yield advantages of the whole cropping system, rather than individual crops, and attributed a significant benefit to maize and a yield reduction in wheat [20]. Therefore, without considering corresponding measures for wheat, the increase in yield of maize by using later-maturing varieties might not offset the yield loss of wheat in future climate change scenarios.

The other genotypic adaptation to increased heat stress can be obtained through improved tolerance of crop pollen viability, e.g., formation of the grain number, to high temperature. Although this study carried out a rough analysis by increasing the threshold for identifying the occurrence of heat stress on grain number from 36 to 37°C, a modelling approach might prove a useful tool to evaluate ex-ante innovative combinations of genes and their interest for future climates to guide breeding approaches [36,37]. In other similar investigations, Gouache et al. [36] showed that using a wheat variety with an increased heat stress threshold during grain filling from 25°C to 26°C would offset the negative effect of climate warming in France. Krishnan et al. [38] showed that using a rice variety with a temperature tolerance threshold for spikelet fertility of 38.5°C rather than 36.5°C could be an effective adaptation strategy in India. Extreme heat is likely to become more frequent with climate warming, and the negative impact of heat stress during the reproductive period has been identified as a major threat to yield in many parts of world [39–41]. Therefore, the potential varietal characteristics mentioned above could be a promising adaptation strategy to avoid food crisis under global warming.

Several important issues could not be addressed in our study. For instance, a single crop model was used for the investigations, which would be plagued with uncertainty [42,43], although the APSIM has proven reliable in the studies. To enhance a more general understanding on assessments of climate change impacts, crop models based on different structures or physiological processes should be included in further work. The use of multiple emission scenarios should also be investigated to cope with uncertainty in simulating crop yield.

## Supporting information

S1 Table Crop data used in this study. (DOCX)
