# Supplementary material for: Ensemble yield simulations: Using heat-tolerant and later-maturing varieties to adapt to climate warming
Source: PLoS One. 2017 May 1;12(5):e0176766. doi: 10.1371/journal.pone.0176766 (PMC5411072; doi:10.1371/journal.pone.0176766)
Supplement: S1 Table — (DOCX) [file pone.0176766.s001.docx]

| Site | Year | Sowing day | Flowering day | Maturity day | Yield(t/ha) |
| --- | --- | --- | --- | --- | --- |
| Yuanyang | 2008 | 166 | 220 | 274 | 10.9 |
|  | 2009 | 167 | 221 | 274 | 9.9 |
| Zhoukou | 2008 | 165 | 220 | 268 | 9.5 |
|  | 2009 | 168 | 218 | 268 | 8.8 |
| Zaozhuang | 2008 | 167 | 217 | 275 | 8.7 |
|  | 2009 | 167 | 217 | 275 | 9.9 |
| Dezhou | 2008 | 169 | 222 | 272 | 8.8 |
|  | 2009 | 167 | 221 | 272 | 8.6 |
| Yongnian | 2008 | 165 | 219 | 275 | 11.0 |
|  | 2009 | 167 | 221 | 275 | 11.4 |
| Shijiazhuang | 2008 | 165 | 219 | 270 | 8.5 |
|  | 2009 | 161 | 216 | 270 | 9.4 |

**S1 Table. Crop data used in this study.**
